# Implementation of an antimicrobial stewardship program targeting residents with urinary tract infections in three community long-term care facilities: a quasi-experimental study using time-series analysis

**DOI:** 10.1186/s13756-015-0095-y

**Published:** 2015-12-01

**Authors:** Sarah B. Doernberg, Victoria Dudas, Kavita K. Trivedi

**Affiliations:** Department of Internal Medicine, Division of Infectious Diseases, University of California, San Francisco, 513 Parnassus Avenue, room S-380, Box 0645, San Francisco, CA 94143 USA; UCSF Medical Center, 505 Parnassus Avenue, San Francisco, CA 94143 USA; Trivedi Consultants, 1563 Solano Avenue, #443, Berkeley, CA 94707 USA

**Keywords:** Urinary tract infection, Antimicrobial stewardship, Long-term care, Antimicrobial resistance

## Abstract

**Background:**

Asymptomatic bacteriuria in the elderly commonly results in antibiotic administration and, in turn, contributes to antimicrobial resistance, adverse drug events, and increased costs. This is a major problem in the long-term care facility (LTCF) setting, where residents frequently transition to and from the acute-care setting, often transporting drug-resistant organisms across the continuum of care. The goal of this study was to assess the feasibility and efficacy of antimicrobial stewardship programs (ASPs) targeting urinary tract infections (UTIs) at community LTCFs.

**Methods:**

This was a quasi-experimental study targeting antibiotic prescriptions for UTI using time-series analysis with 6-month retrospective pre-intervention and 6-month intervention period at three community LTCFs. The ASP team (infectious diseases (ID) pharmacist and ID physician) performed weekly prospective audit and feedback of consecutive prescriptions for UTI. Loeb clinical consensus criteria were used to assess appropriateness of antibiotics; recommendations were communicated to the primary treating provider by the ID pharmacist. Resident outcomes were recorded at subsequent visits. Generalized estimating equations using segmented regression were used to evaluate the impact of the ASP intervention on rates of antibiotic prescribing and antibiotic resistance.

**Results:**

One-hundred and four antibiotic prescriptions for UTI were evaluated during the intervention, and recommendations were made for change in therapy in 40 (38 %), out of which 10 (25 %) were implemented. Only eight (8 %) residents started on antibiotics for UTI met clinical criteria for antibiotic initiation. An immediate 26 % decrease in antibiotic prescriptions for UTI during the ASP was identified with a 6 % reduction continuing through the intervention period (95 % Confidence Interval ([CI)] for the difference: −8 to −3 %). Similarly, a 25 % immediate decrease in all antibiotic prescriptions was noted after introduction of the ASP with a 5 % reduction continuing throughout the intervention period (95 % CI: −8 to −2 %). No significant effect was noted on resistant organisms or *Clostridium difficile*.

**Conclusion:**

Weekly prospective audit and feedback ASP in three community LTCFs over 6 months resulted in antibiotic utilization decreases but many lost opportunities for intervention.

## Background

Asymptomatic bacteriuria in the elderly commonly results in antibiotic administration despite evidence showing no clinical benefit [1–6]. In turn, antibiotic overuse contributes to antimicrobial resistance, adverse drug events, and increased costs in the long-term care facility (LTCF) population [7–17]. As the elderly population grows, these consequences become more problematic.

Currently, over 3 million individuals reside in LTCFs in the United States, and the complexity of underlying conditions is increasing [18]. Correspondingly, these residents frequently transition to and from the acute-care setting, often transporting drug-resistant organisms across the continuum of care [19, 20]. Recently published work assessing prevalence of drug-resistant organisms in LTCFs indicates that antimicrobial stewardship efforts are necessary [21, 22].

Antimicrobial stewardship programs (ASP) are viewed as resident safety initiatives designed to improve clinical outcomes while reducing adverse effects [23]. These types of programs have proven to be effective in acute care hospitals and LTCFs affiliated with tertiary care centers but have not been as well-studied in community LTCFs [24–34]. Because staffing levels, patient care models, and overall goals differ between the LTCF and the acute care settings, the ideal design and execution of an ASP in the LTCF setting will likely diverge. In addition, since September 2009 when the Interpretative Guidelines for Long-Term Care Facilities was issued, the Centers for Medicare and Medicaid Services (CMS) have stated that it is the LTCF physician’s responsibility to prescribe appropriate antibiotics and to establish the indication for use of these medications; furthermore, consultant pharmacists are encouraged to review indications for antibiotic use and report findings to the physician [35]. Despite this regulatory backbone, it remains unclear how best to implement ASPs in the LTCF setting.

The objectives of this study were to assess the feasibility and efficacy of implementing an ASP utilizing a syndromic approach targeting urinary tract infections (UTIs) at community, stand-alone LTCFs.

## Methods

### Study design

A prospective quasi-experimental study was performed to implement an ASP targeted at UTIs diagnosed and treated at three community LTCFs in Northern California between September 2011 and May 2012. The ASP team consisted of an Infectious Diseases (ID)-trained clinical pharmacist and an ID physician, who worked closely with the infection control practitioners at each of the respective LTCFs.

The study period was divided into two phases: pre-intervention (7 months) and intervention (7 months). During the pre-intervention phase, baseline information on facility-level antimicrobial susceptibility patterns and antimicrobial utilization were collected from each LTCF. During the intervention phase, the ID pharmacist made weekly site visits to each LTCF to identify residents receiving antibiotics for UTIs which was determined by infection control and nursing administration records at each site. Individual variables, including resident demographics, comorbidities, vital signs, documented exam findings, laboratory results, and additional antibiotics for each resident on antibiotics for UTI, were collected weekly by the ID pharmacist by review of the medical record. The ID pharmacist and ID physician then consulted, and recommendations were formulated utilizing the Loeb clinical consensus criteria for initiation of antibiotics in the LTCF setting as a guideline [36]. For residents not meeting clinical consensus criteria, the ASP team used clinical judgement including input from subspecialists and the resident’s predisposition for other infections, to determine if antibiotics were indicated and to help formulate recommendations. The ID pharmacist subsequently conveyed the ASP recommendations to the primary treating provider via telephone or fax. Fax was utilized a minority of the time with one specific provider who expressed a preference for this form of communication. Implementation of recommendations and clinical course of each resident was recorded at subsequent visits, including vital sign abnormalities, white blood cell count, change in antibiotics, need to transfer to acute care, or death. Data on facility-level antibiotic susceptibility patterns and antibiotic utilization was collected, in a similar manner to the pre-intervention phase, for the intervention phase.

### Ethics, consent, and permissions

This study was reviewed by the Committee for the Protection of Human Subjects of the California Health and Human Services Agency and was deemed not to be research and therefore exempt from approval. As a result and because implementation of this study was evaluated as a quality improvement initiative, consent was not obtained from residents in the participating facilities.

### Setting and population

This intervention took place at three community, stand-alone LTCFs in Northern California: facility A is licensed for 77 subacute beds and 82 skilled nursing beds with 3 physicians; facility B has 478 licensed skilled nursing beds with 5 physicians and 1 nurse practitioner; facility C is comprised of two sister facilities located blocks away from each other and licensed for 60 and 65 skilled nursing beds respectively with 2 physicians and 1 nurse practitioner. Because the medical staff and population of these latter two facilities overlap significantly, facility C was analyzed as one LTCF. Subjects included any resident of the skilled nursing or subacute sections of these LTCFs being treated for UTI with an antibiotic at the time of the ID pharmacist visit each week. Subjects were excluded if they resided in the psychiatric or acute rehabilitation units at each facility or if antibiotic initiation for UTI occurred at an acute care facility.

### Process and outcome measurements

Outcome of recommendations and clinical course for residents experiencing ASP interventions were recorded at subsequent visits through chart review. Recommendations were considered “accepted” if the suggested change (or discontinuation) of antimicrobials was made within 24 h of the ASP recommendation being communicated. Resident-days were collected from each facility. Antibiotic use was measured as antibiotic starts per 1000 resident-days. Data on antibiotic starts were obtained from the infection control practitioner at each facility. All LTCFs utilized a maintenance log of all antibiotic starts, which included indication for the antibiotic, allowing for determination of both a UTI and overall rate of antibiotic use.

Culture data, including information on susceptibility, was obtained from the diagnostic laboratories utilized by each of the facilities. As a marker for potential downstream consequence of antibiotic pressure, we collected information on rates of *Clostridium difficile*, ceftriaxone-resistant *Enterobacteriaceae* (including extended-spectrum β-lactamase (ESBL) producing organisms), fluoroquinolone-resistant *Pseudomonas aeruginosa*, and vancomycin-resistant Enterococci isolated from any site for each facility. Rates of these organisms were calculated from cultures that were collected as a part of routine clinical care. An individual resident could contribute more than one clinical culture since the unit of measurement was the culture. Rates were calculated based on number of cases of each resistant organism normalized to resident-days.

### Analysis

Generalized estimating equations using segmented regression and a Poisson distribution accounting for clustering by facility were used to evaluate the impact of the ASP intervention on rates of antibiotic prescribing and antibiotic resistance. This model generated four important estimates: 1. The pre-intervention trend in incidence (β1); 2. The immediate change upon initiation of the ASP (β2); 3. The difference between the pre- and intervention trend (β3); and 4. The intervention period rate change (β1+ β3) [37]. The combination of β1 and β3 prevented the attribution of changes preceding the intervention from being attributed to the effects of the intervention. Because of the concern for autocorrelation between observations over the time of this study given the time-series design, first order positive and negative autocorrelation was assessed using the Durbin-Watson statistic, which did not suggest evidence of autocorrelation in these models. Robust standard errors were used to estimate variance. Significance was defined as *P* ≤ 0.05. STATA software (ver 11, StataCorp 2009) was used for all statistical analyses except the Durbin-Watson statistic, which were performed with SAS version 9.3 (SAS Institute Inc., Cary, NC).

## Results

### Resident characteristics

During the historical pre-intervention phase (April 2011–October 2012), there were combined 118,070 resident-days from the participating LTCFs. During the intervention phase (November 2012–May 2012), there were combined 113,220 resident-days from the LTCFs. Table 1 demonstrates the baseline characteristics of the 104 reviewed prescriptions during the intervention. The average temperature and white blood cell counts for residents were within the normal range. Most residents had pyuria and positive markers of urinary tract inflammation on urinalysis. Despite these findings, only 8 % of residents started on antibiotics for UTI who were evaluated during the intervention met the Loeb minimum criteria for antibiotic initiation [36]. Of those meeting Loeb criteria, 6 % were not catheterized and 2 % catheterized. Both of the catheterized residents met Loeb criteria on fever alone. In those non-catheterized residents meeting criteria, 33 % had dysuria plus urinary frequency, 33 % had dysuria plus incontinence, 17 % had fever plus hematuria, and 17 % had fever plus incontinence.

**Table 1:** Characteristics of residents started on antibiotics for urinary tract infection who were reviewed during the intervention

Baseline characteristic	Intervention (*n* = 104)
Age, mean, years (Standard Deviation (SD))	80.8 (14.4)
Male gender, number (%)	70 (67)
Facility, number (%)	–
A	22 (21)
B	63 (61)
C	19 (18)
Charlson comorbidity index, median (IQR)	2 (1–3)
Temperature, mean, F (SD)	98.2 (1.1)
Indwelling catheter in prior 48 h, number (%)	7 (7)
WBC × 10^9^/L, mean (range)	9.9 (4.1–21.3)
Urine WBCs/hpf >10, *n* (%)	89 (86)
Urine leukocyte esterase, number/total sent (%)	89/91 (98)
Urine nitrites, number/total sent (%)	51/91 (56)
Meets criteria for UTI, *n* (%)	8 (8)
No catheter	6 (75)
Catheter	2 (25)

*IQR* interquartile range, *WBC* white blood cell count, *hpf* high-powered field, *SD* standard deviation, *UTI* urinary tract infection

*Escherichia coli*was the most common urinary organism treated (in 70 % of residents), and gram-negative rods predominated the positive urine cultures (accounting for 95 % of treated urine cultures). Of the prescriptions that were reviewed, fluoroquinolones were the most commonly prescribed antibiotics (9 prescriptions, 39 %), followed by nitrofurantoin (5 prescriptions, 22 %), trimethoprim-sulfamethoxazole (4 prescriptions, 17 %), cephalexin (3 prescriptions, 13 %), and amoxicillin +/− clavulanate (2 prescriptions, 9 %). Intravenous therapy was infrequently prescribed.

### Process measures

There were 292 prescriptions for UTI during the pre-intervention phase and 183 during the intervention. Of the 183 prescriptions for UTI, 104 were able to be reviewed by the Pharmacist. Of these, recommendations for change in therapy were made in 38 %, and 10 (25 %) were accepted. Twenty-four percent of the recommendations were to discontinue antibiotics; 2 % to streamline antibiotics; and 11 % to shorten the course of antibiotics. No recommendations were made to broaden antibiotics, lengthen course, or change route. The majority of recommendations were made by phone. A small minority (<5 %) were made by fax.

Of the 59 % of antibiotic prescriptions for UTI where recommendations were not communicated to the primary provider, there was either agreement with current management (12 residents, 19 %) or completion of the antibiotic course within 2 days from the time of review (52 residents, 81 %). Because the antimicrobial course was due to end concurrently with review, interventions were not made in the latter group. Of this group of 52 residents, antibiotics were not felt to be indicated in 44 (85 %). Of note, the percentage of residents where there was concordance with antibiotic management exceeded the percentage meeting Loeb criteria for UTI. In the cases where there was a discrepancy, the ASP team considered extenuating circumstances, including recommendations for antibiotics from other subspecialists, family dynamics, and concurrent treatment for other infections. The remainder of antibiotic prescriptions was not reviewed due to an inability to determine if the prescription was for UTI, the entire antibiotic course was completed in between weekly ID Pharmacist visits, or the antibiotic was initiated in an acute care setting.

### Outcome measures

**Fig. 1:**
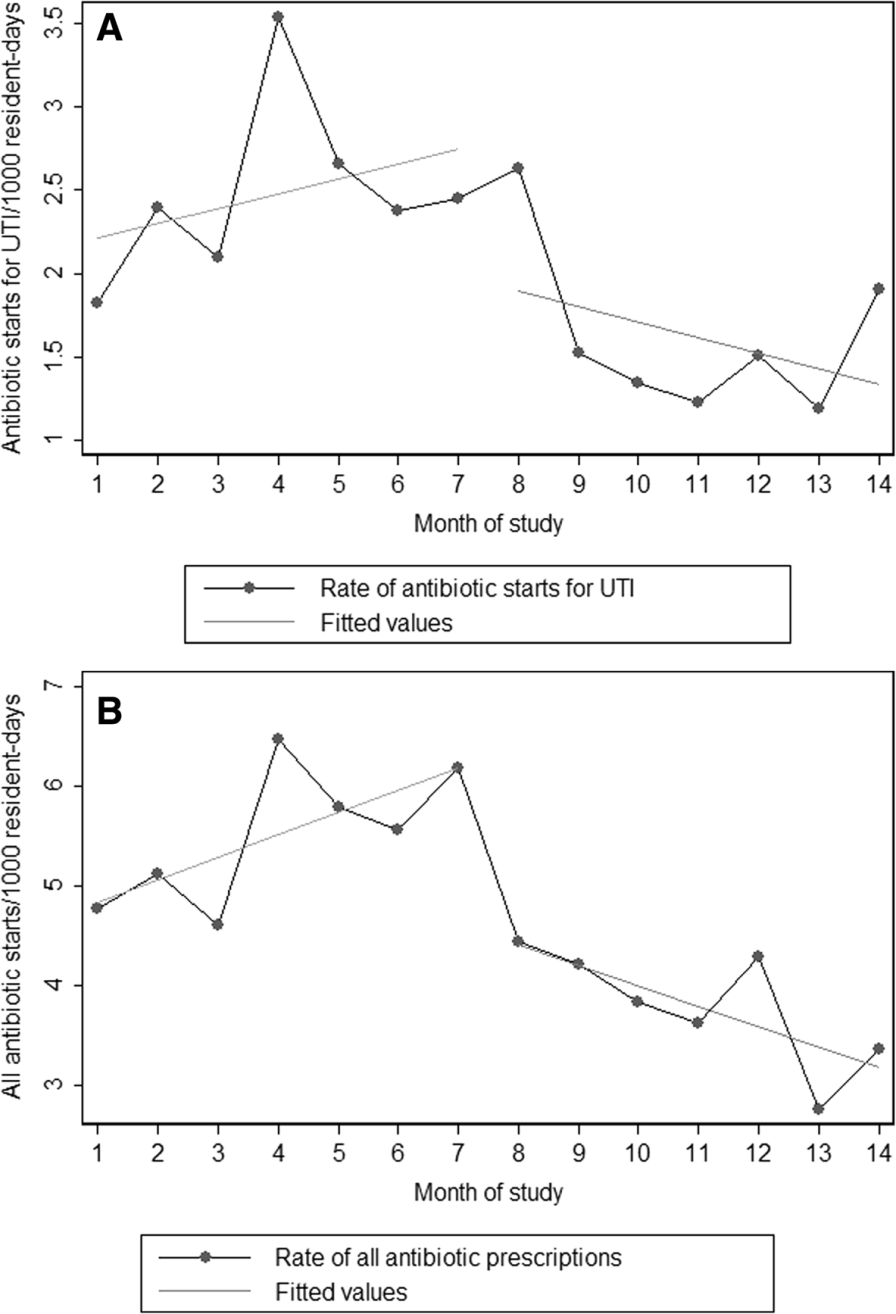
**a** Predicted and actual rates for antibiotic starts for UTI per 1000 resident-days; (**b**) Predicted and actual rates for antibiotic starts for all indications per 1000 resident-days. Predicted rates are based on the intervention trend calculated from the time-series model. The intervention started in month 8

Crude incidence rates for antibiotic prescriptions and resistant organisms are shown in Table 2. Incidence rate ratios for the segmented regression for antibiotic utilization are shown in Table 3. Monthly rates, both measured and predicted by our statistical model, for antibiotic starts are shown in Fig. 1. During the pre-intervention phase, there was a trend towards a significant increase in antibiotic starts for UTI (4 % increase, *P* = 0.06). Upon initiation of the ASP intervention, a 26 % immediate decrease in antibiotic prescriptions for UTI was observed, and there was a 9 % change in the trend from the pre-intervention phase to the intervention phase. After the initial intervention effect, there was a 6 % decrease in the rate of antibiotic prescriptions for UTI per month continuing throughout the intervention period (95 % Confidence Interval [CI]: −8 to −3 %). There was no change in antibiotic prescription rates for all indications during the pre-intervention phase. With the introduction of the intervention, there was a 25 % decrease in all antibiotic prescriptions. After this initial decrease, there was a non-significant trend toward decreasing antibiotic prescriptions compared to the pre-intervention phase (95 % CI: −19 to 2 %). During the entire intervention phase, there was a 5 % decrease in all antibiotic starts.

**Table 2:** Crude incidence rates for antibiotic starts

Month	Antibiotic starts, UTI^a^	Antibiotic starts, all indications
1	1.8	4.8
2	2.4	5.1
3	2.1	4.6
4	3.5	6.5
5	2.7	5.8
6	2.4	5.6
7	2.4	6.2
Intervention began		
8	2.6	4.4
9	1.5	4.2
10	1.3	3.8
11	1.2	3.6
12	1.5	4.3
13	1.2	2.8
14	1.9	3.4

*UTI* urinary tract infection

^a^N/1000 resident-days for antibiotic measurements

**Table 3:** Incidence rate-ratios for an interrupted time-series model of antibiotic prescriptions

Parameter	Co-efficient	Incidence rate ratio (95 % confidence interval)	*P*-value
		Antibiotic starts, UTI	
Pre-intervention trend	β1	1.04 (1.00–1.07)	0.06
Immediate intervention change	β2	0.74 (0.64–0.84)	<0.001
Change in trend after intervention	β3	0.91 (0.89–0.93)	<0.001
Intervention trend	β1 + β3	0.94 (0.92–0.97)	<0.001
		All antibiotic starts	
Pre-intervention trend	β1	1.04 (0.96–1.13)	0.30
Immediate intervention change	β2	0.75 (0.67–0.84)	<0.001
Change in trend after intervention	β3	0.91 (0.81–1.02)	0.09
Intervention trend	β1 + β3	0.95 (0.92–0.98)	0.001

To evaluate the consequence of antibiotic pressure on clinical cultures for common resistant organisms, rates of *Clostridium difficile*, ceftriaxone-resistant *Enterobacteriacaeae*, fluoroquinolone-resistant *Pseudomonas aeruoginosa*, and vancomycin-resistant *Enterococci* were examined. There were no significant changes in rates of any of these organisms throughout the study period (data not shown).

## Discussion

Introduction of a weekly prospective audit and feedback ASP in three community LTCFs resulted in modest decreases in antibiotic utilization but encountered several barriers to effective ASP implementation. Despite having a dedicated ID pharmacist and physician available once weekly for reviews, there were many missed opportunities for intervention and low acceptance rates when recommendations were made.

Given the modest effect observed with implementation of our ASP in this study, we have identified several areas for improvement in future interventions. First, given that 85 % of the prescriptions that were not reviewed were deemed unnecessary, an ASP with more frequent review of residents on antibiotics might be more useful, though it may not be feasible with resource limitations and the general unavailability of ID physicians and pharmacists in the LTCF setting. More education and training of non-Infectious Diseases specialized providers may help to bolster a more robust ASP at these facilities.

Second, there were many missed opportunities for intervention to improve or to identify prescribing habits associated with good antimicrobial stewardship, even if the recommendation would not affect that particular course of antimicrobials (e.g. in the case of a resident whose course was scheduled to end the day of review). In future studies, educational feedback with the goal of broadly changing antimicrobial prescribing habits may be more successful. The large initial decrease in antimicrobial prescriptions suggests that knowledge of program implementation itself may have affected prescribing practice; whether this decrease would have been sustained longer than 6 months warrants further investigation.

In addition, our largest barrier was establishing relationships with prescribers in this setting as compared with acute care. Because much of the medical care occurs remotely in the LTCF population, establishing interpersonal relationships between the ASP and the primary treating providers proved challenging due to lack of face-to-face interaction and lack of a prior provider-to-provider relationship. This barrier limited physician and advanced care practitioner buy-in and implementation of recommendations. In future interventions, it may be helpful if the ASP champion was identified from within the institution, though this may be problematic if sufficient knowledge of antimicrobial stewardship principles is lacking at the local level. If stewardship is to be initiated by an outside consultant, concerted efforts to establish a relationship with providers should be pursued, such as educational seminars, face-to-face meetings, and collaboration in design of the program.

Lastly, primary treating providers reported feeling pressured by nursing staff and, to some extent, resident families to send urinalyses and urine cultures for indications such as cloudy urine, foul-smelling urine, or temporary behavior changes. When these cultures tested positive, regardless of whether the symptoms had disappeared prior to therapy, the residents were often treated. Front-line staff education must remain a specific focus of future ASP interventions in the LTCF setting since this group plays a vital role in establishing the prescribing culture of an institution. A recent study at the VA Healthcare System, which included an associated long-term care facility, found that an educational campaign aimed to improve treatment for catheter-associated UTI decreased the number of urine cultures sent by 71 %, which corresponded to a 76 % decrease in overtreatment for asymptomatic bacteruria [38]. The decreases were most pronounced in the LTCF portion of the study. Stewardship of urinalysis and urine culture, including among nursing staff and families, may be an effective upstream method for decreasing inappropriate antibiotic use for UTI in the LTCF setting. In fact, in the acute care setting, a recent pilot study demonstrated that suppressing urine culture results in noncatheterized patients resulted in decreased treatment of asymptomatic bacteriuria without any untoward consequences [39]. Though this degree of intervention may be risky and require more resources, it does support the notion that limiting cultures may limit overtreatment of asymptomatic bacteriuria.

Our study has several additional important limitations aside from the barriers to success. Because it took place over a 14-month period, the pre-intervention and intervention phases occurred during different seasons, so temporal trends could not be eliminated. We attempted to adjust for this with our statistical analyses by using a time-series analysis approach. In addition, the 2011–2012 influenza season was accounted for mainly during the intervention phase, so antibiotic prescriptions would be expected to increase during the intervention, which is the opposite of what was observed. We only identified 104 cases of treated UTI in three nursing homes in 6 months of our study intervention so our study power was lower than expected but sometimes unavoidable in real-life research. In order to identify cases we relied on Infection Control reports of antimicrobial prescriptions, which may have been limited by recall bias. Obtaining prescription data would have allowed for a more objective measurement of prescriptions during the pre- and post-intervention periods. It would also have allowed for more information regarding days of therapy and length of therapy. Unfortunately, we did not have access to pharmacy records at two of the sites due to pharmacies being located off-site and operated by third-party vendors.

Several other groups have implemented stewardship initiatives in the LTCF setting with varied success. Jump and colleagues found an even larger decrease in antimicrobial utilization with initiation of an ID service at a LTCF associated with a Veterans Affairs Hospital [40]. That study included a more resource-intensive and comprehensive approach to antimicrobial stewardship with a team that physically staffed consults during weekly visits and took additional calls throughout the week, a model that is unlikely to be feasible, especially at LTCFs that are not academically affiliated. Several other studies have explored less resource-intensive approaches, such as educational interventions. Two studies have demonstrated approximately 30 % decrease in antimicrobial prescribing for UTI after educational interventions aimed at appropriate evaluation and treatment of UTI versus asymptomatic bacteriuria [29, 33]. Additional studies have demonstrated 12–30 % decreases in antibiotic use with educational interventions focused on appropriate diagnosis and treatment of common infectious syndromes [31, 32]. The decrease in antibiotic use in our study was lower than that seen with the educational interventions alone. However, the most effective published strategy is an educational intervention consisting of mailing an antibiotic prescribing guide combined with physician-specific antibiotic prescribing profiles (physicians in the experimental group were 64 % less likely to prescribe non-adherent antibiotics than those in the control group (Odds Ratio = 0.36, 95 % CI = 0.18–0.73), suggesting that physician feedback is an essential component [30]. Provider characteristics have been demonstrated to play an important role in duration of antibiotic prescriptions independent of severity of underlying disease, suggesting that high-prescribing physicians may be good targets for these types of ASP interventions [41]. Combining education with an audit and feedback ASP would allow for targeted physician feedback and therefore even more benefit on improving prescribing habits.

## Conclusion

Our findings suggest that an ASP with a syndromic approach has the potential to be effective in the LTCF setting but further studies are needed to determine the most robust and efficient design for such an intervention. The addition of an educational program with prescriber feedback, which has proven beneficial in prior studies, might strengthen the benefits of an audit and feedback ASP alone. Our ASP was not able to provide as much educational support given that the ASP team and primary treating providers were not often at the LTCF at the same time. A dedicated educational aspect to the ASP would also allow for institutional changes such as educating the nursing staff in identifying residents at risk and diagnosing bacteriuria as well as the conviction from providers that positive cultures mandate therapy. Moving forward, qualitative analyses may be important to identify key stakeholders in the antibiotic prescribing process in LTCFs. Design of future interventions should incorporate education targeting these stakeholders as well as focused audit and feedback of prescriptions for specific common infectious syndromes.
